# Investigating Synthetic Oligonucleotide Targeting of Mir31 in Duchenne Muscular Dystrophy

**DOI:** 10.1371/currents.md.99d88e72634387639707601b237467d7

**Published:** 2016-06-16

**Authors:** John CW Hildyard, Dominic J Wells

**Affiliations:** Department of Comparative and Biomedical Sciences, The Royal Veterinary College, London, UK; Department of Comparative and Biomedical Sciences, The Royal Veterinary College, London, UK

## Abstract

Exon-skipping via synthetic antisense oligonucleotides represents one of the most promising potential therapies for Duchenne muscular dystrophy (DMD), yet this approach is highly sequence-specific and thus each oligonucleotide is of benefit to only a subset of patients. The discovery that dystrophin mRNA is subject to translational suppression by the microRNA miR31, and that miR31 is elevated in the muscle of DMD patients, raises the possibility that the same oligonucleotide chemistries employed for exon skipping could be directed toward relieving this translational block. This approach would act synergistically with exon skipping where possible, but by targeting the 3’UTR it would further be of benefit to the many DMD patients who express low levels of in-frame transcript. We here present investigations into the feasibility of combining exon skipping with several different strategies for miR31-modulation, using both in vitro models and the mdx mouse (the classical animal model of DMD), and monitoring effects on dystrophin at the transcriptional and translational level. We show that despite promising results from our cell culture model, our in vivo data failed to demonstrate similarly reproducible enhancement of dystrophin translation, suggesting that miR31-modulation may not be practical under current oligonucleotide approaches. Possible explanations for this disappointing outcome are discussed, along with suggestions for future investigations.

## Introduction

Duchenne muscular dystrophy (DMD) is an X-linked muscle-wasting condition caused by low or absent expression of the muscle protein dystrophin, leaving muscle fibres exquisitely vulnerable to exercise-induced damage (particularly eccentric exercise). The condition is characterized by repeated cycles of muscle degeneration and regeneration, leading to progressive muscle wasting and accumulation of fibrosis and fatty deposits. DMD is invariably fatal, and no current cure exists: existing therapies are chiefly concerned with minimizing inflammatory damage (corticosteroid treatment regimes^1^), providing respiratory assistance with positive pressure ventilation^2^, and using drugs to treat the cardiomyopathy^3^, and do not address the primary defect (insufficient/absent dystrophin protein). While a number of additional avenues are being explored, including anti-fibrotic agents^4^, promotion of muscle hypertrophy^5^^,^ 6, and modulation of muscle metabolism^7^, the core focus of research remains the restoration of dystrophin expression.

The dystrophin gene is huge; at around 3Mb (and comprised of 79 exons), this gene occupies roughly 0.1% of the entire human genome. This gene is transcribed and spliced into an mRNA roughly 14,000 bases in length, and ultimately translated to a protein 427kDa in size. The dystrophin protein has a barbell-like structure, with the actin-binding N-terminus and the dystroglycan/nNOS binding C-terminus linked by an extended stretch of 24 spectrin-like repeats^8^^,^ 9. Becker’s muscular dystrophy (BMD), in most cases a considerably milder -and sometimes largely asymptomatic- dystrophic condition, typically results from mutations causing deletions of this internal repeat region: all crucially retaining the reading frame. While several functional elements are located within this linker region (including an additional actin-binding domain^10^ and an nNOS localisation motif^11^), as long as functional N and C terminal domains are expressed and remain linked, the full extent of the central linking domain is not absolutely critical to dystrophin function. This observation underpins many dystrophin-restoring therapies where restoring full-length dystrophin would otherwise be technically prohibitive: from microdystrophin therapies (plasmid or viral delivery of a substantially internally-truncated dystrophin)^12^^,^ 13 ^,^ 14 to exon-skipping approaches (using short synthetic antisense oligonucleotides to alter splicing patterns, ‘skipping’ exons to restore reading frame, generating an internally-deleted but functional dystrophin)^15^^,^ 16 ^,^ 17 ^,^ 18 ^,^ 19. Candidates from this latter category, using both 2-O’methyl phosphorothioate and phosphorodiamidate morpholino oligomers (2Ome and PMO, respectively) are currently at the late clinical trial stage, though the efficacy of this approach is limited by the extent of oligonucleotide delivery, and by the requirement for sequence-specific targeting. A wide range of mutations to the dystrophin gene can result in a DMD phenotype, necessitating skipping of a range of specific exons. Even the current generation of oligos, designed to restore the reading frame to multiple mutations in a “hot spot” on the dystrophin gene by excluding exon 51, are expected to be of use to only 13% of DMD patients^20^. Therapies capable of enhancing dystrophin expression in a less mutation-specific fashion would thus be of considerable advantage.

The discovery that the translation of dystrophin transcripts is subject to negative regulation by the microRNA miR31, and that this microRNA is dysregulated in dystrophic muscle^21^^,^ 22 ^,^ 23, suggested that inhibition of miR31 might constitute a therapeutic approach.

MicroRNAs (miRs) are produced as ca. 200 nucleotide “pri-microRNAs” which undergo several discrete processing steps to generate first “pre-microRNAs”, and ultimately mature microRNAs (see schematic: fig 1 A)^24^.

**miR processing, canonical mode of action and miR-modulation scheme:**
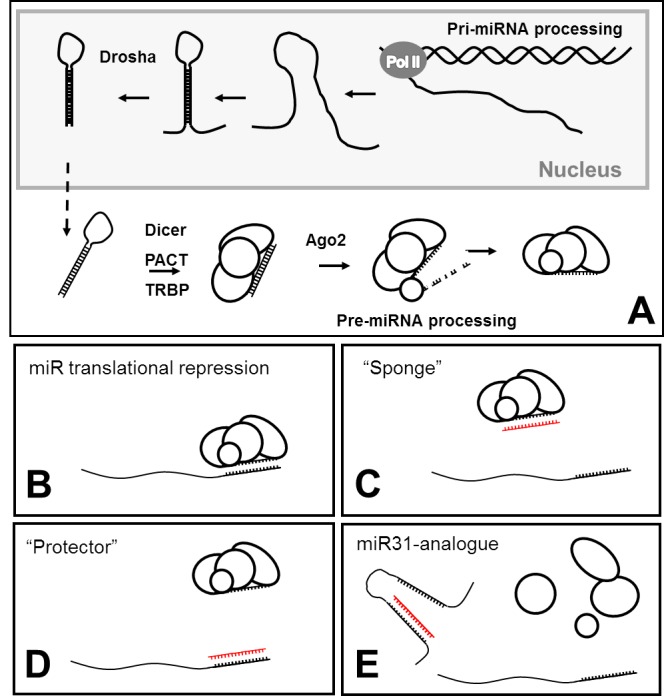
MicroRNAs are transcribed as pri-miRs, processed to pre-miRs in the nucleus and finally processed to mature miRs in the cytosol, remaining associated with several proteins in a silencing complex (A). miR-mediated translational suppression requires interaction of microRNA with the 3’UTR of transcripts (B), and can potentially be inhibited by sequestration of miR (Sponge, C), masking of the binding site on 3’UTR (protector, D) or by interfering with miR maturation (analogue, E).

MiR31 is highly expressed in satellite cells (the muscle stem cell population) where under healthy physiological conditions it is believed to suppress translation of transcripts for early myogenic factors such as myf5^25^, allowing relatively high levels of mRNA to accumulate without eliciting a commitment to the myogenic program. Following satellite cell activation, levels of miR31 fall as myogenesis progresses, in a process believed to be nitrosylation-sensitive^26^: localisation of nNOS to the sarcolemma by dystrophin alters nuclear nitrosylation patterns, lowering miR31 expression and thus enhancing dystrophin levels, potentially forming a feed-forward loop generating a switch-like commitment to terminal myogenesis. In the absence of sufficient dystrophin no loop can be established and cells remain trapped at a late but incomplete stage of differentiation.

Blocking the action of miR31 would therefore be expected to increase translational competence of any given dystrophin transcript, giving effectively greater protein expression per mRNA molecule. Moreover, if the feed-forward hypothesis is correct, enhancement of translation might exhibit threshold effects: above a certain level of dystrophin the suppression of miR31 may become self-sustaining. A final appealing aspect of this strategy is that the binding site for miR31 resides in the 3’UTR of the dystrophin transcript and is not associated with reading frame or coding mutations. It should thus be common to all patients, and a miR31-based therapy would be synergistic with exon-skipping approaches regardless of the targeting of the skipping oligo.

Work by Cacchiarelli *et al* 21 showed that modulation of miR31 activity, either by a “sponge” approach (using an mRNA carrying multiple partially-complementary miR31 binding sites to sequester active miR31 without being targeted for degradation) or by “protector” approach (using an antisense locked nucleic acid –LNA- to mask the miR31 binding sequence on the dystrophin transcript, preventing interaction of miR31 with this mRNA) enhanced expression of dystrophin at the protein level in dystrophic cells in culture, suggesting that this represents a viable therapeutic avenue.

In this study we therefore determined to investigate whether blockade of miR31 could be employed synergistically with exon-skipping, using PMO oligonucleotide chemistries already approved for use in clinical trials. Using an animal model of DMD, the *mdx* mouse (which carries a premature stop codon in dystrophin exon 23), and a dystrophic mouse cell culture line carrying the *mdx* mutation (H^2K^SF1^27^), we combined the well-established *mdx*-specific skipping oligo (M23D)^28^ with PMOs representing “sponge”, “protector” and “miR31-analogue” sequences (schematic: Fig 1 B-E, sequences and hybridization scheme in supplementary fig S1).

As inhibition of miR31 would be expected to increase total “protein per transcript”, we measured (to high precision) the levels of both successfully-skipped dystrophin mRNA and of dystrophin protein, thus expressing efficacy of miR31 modulation as a protein:mRNA ratio

## Materials and methods

*Mdx* mice were on a C57BL10ScSn background. Animal experiments were carried out under license from the Home Office (UK) in accordance with The Animals (Scientific Procedures) Act 1986 and were approved by the Royal Veterinary College ethics and welfare committee. Mice were housed in a minimal disease facility in cages of 3-5 individuals, with food and water *ad libitum.*

All reagents were purchased from Sigma/Fluka unless indicated otherwise.

**Cell Culture**

The H^2K^SF1 cell line (derived from an *mdx*-immortomouse cross) were grown in matrigel-coated flasks (0.1mg.ml-1) and maintained in a proliferative state by incubation at 33°C in proliferation medium: DMEM+glutamax (invitrogen) supplemented with 20% (v/v) heat inactivated-foetal bovine serum, 2% (v/v) chicken embryo extract (CEE, Sera laboratories international), 1% (v/v) Penicillin/Streptomycin (Sigma, final concentration 100u.ml-1 penicillin, 100ug.ml-1 streptomycin), and 20 U/mL γ-IFN (Chemicon).

One day prior to differentiation, cells were seeded onto matrigel-coated 6-well plates at 5x10^5^ cells.well^-1^; plating conditions established to allow optimal differentiation without under- or overcrowding cells. Differentiation was initiated by replacement of proliferation media with differentiation medium (DMEM+glutamax supplemented with 5% horse serum (PAA) and 1% pen/strep) and incubation at 37°C. Differentiation medium was partially replaced (50% of medium aspirated, replaced with fresh differentiation medium) after 2 days of differentiation.

For naked PMO/Vivo-Morpholino treatments, oligos (1.3uM final) were added directly to the culture medium 48 to 52 hours after initiation of differentiation, and gently swirled to mix. For staggered administrations additional oligos were added after a further 24 or 48 hours. No further media changes were performed after oligo addition.

For nucleofections, 1x10^6^ proliferating cells were incubated in 100ul nucleofection solution supplemented with PMOs as indicated and nucleofected using protocol B32 (using a nucleofector 2B –Lonza), before dilution into 2ml proliferation media and seeding onto a 6-well plate. Cells were subsequently differentiated as described above.

Both protein and RNA were isolated from the same culture wells using the following protocol: culture media was aspirated gently to avoid detaching myotubes, and cells were lysed by addition of 100ul chilled RIPA buffer (50mM Tris pH8, 150mM NaCl, 1% NP40, 0.5% sodium deoxycholate, 0.1% SDS, supplemented with protease inhibitors –complete, Roche) then detached rapidly by agitation with a cell scraper before removal of a 50ul aliquot directly into 100ul of TRIzol reagent for RNA extraction. The remaining 50ul RIPA lysate was maintained on ice for 10min before centrifugation in a benchtop microfuge to remove insoluble myofibrillar material. Both RNA and protein isolates were stored at -80°C until needed.

***In vivo* studies**

*mdx* mice were treated with morpholinos (in saline solution) as follows:

PMO: 5ug M23D skipping oligo either alone or supplemented with protector, 31 analogue (5ug) or sponge (20ug), in a final volume of 20ul, was delivered via a single direct intramuscular (IM) injection into the tibialis anterior (TA) muscle.

Vivo-Morpholino: M23D Vivo-Morpholino was delivered by a single intravenous (IV) injection into the tail vein at 5mg.kg^-1^ (ca. 200ug.mouse^-1^, 180-200ul final volume), or via a single direct IM injection to the TA muscle at 10ug.mouse^-1^ (20ul final volume), or via a single intraperitoneal (IP) injection (5mg.kg^-1^ M23D either alone or combined with 5mg.kg^-1^ protector or scrambled Vivo-Morpholino, 180-200ul final volume).

All IM injections were performed under hypnorm/hypnoval anaesthesia and IV injections under isoflurane. Anaesthetised mice were allowed to recover in a heated chamber. All mice treated intravenously with Vivo-Morpholinos died within minutes of injection (see results) thus this treatment was discontinued.

Two weeks post-injection mice were killed by cervical dislocation and tissues collected rapidly (as described below) for histology and RNA/protein analysis.

Vivo-Morpholino IP-treated mice: diaphragms and body wall sections were removed and halved: one half used for histology, the other for RNA/protein isolation. Histological samples were rolled up and mounted vertically on cork blocks, coated in cryogenic mounting medium (cryo-M-bed, bright) and frozen rapidly in liquid nitrogen-cooled isopentane to preserve tissue morphology. Tissues were cryosectioned into 10 micron sections and mounted on glass slides (Superfrost, fisher scientific), with serial sections collected and mounted every 500um through the sample, permitting assessment of the entire tissue bulk. For RNA/protein isolation, samples were flash frozen and pulverised under liquid nitrogen.

PMO IM-treated mice: injected TA muscles were mounted vertically on cork blocks free of cryogenic mounting and frozen under liquid nitrogen-cooled isopentane and serially cryosectioned as above. The omission of mounting medium allows intervening sections to be collected and used for RNA/protein analysis.

**Histology**

Muscle cryosections (10 micron thickness) were stained for dystrophin essentially as described by Wells *et al* 19, using antibodies raised to the dystrophin C-terminal domain (rabbit polyclonal DysC3750, Cymbus Biotechnology, 1:500) with biotinylated secondary antibodies (biotinylated goat anti-rabbit, DAKO, 1:500). Signal was developed using the ABC kit system (VECTASTAIN) with diaminobenzedine (SIGMAFAST) for 3 mins per slide. For IM-injected muscle, dystrophin-positive fibres were counted, while for Vivo-Morpholino treated muscle samples were assessed by eye and densitometry (using ImageJ). In both cases samples were assessed blind to sample identity.

**Western Blotting**

RIPA-solubilized cell cultures and muscle sections (typically 30-40 10um sections) were used for western blotting essentially as described by Godfrey *et al* 29, using hand-cast 4% acrylamide gels and slow wet-blotting onto PVDF membranes. Blots were probed with anti-dystrophin antibody 6D3 (millipore, 1/200 for tissue sections, 1/100 for cell culture) and anti-vinculin h-Vin1 (sigma, 1/100,000 for tissue sections, 1/250,000 for cell culture), using anti-mouse HRP (BioRad, 1/100,000) as secondary. Blots were developed using ECL prime (Amersham).

**RNA extraction & cDNA synthesis**

RNA extractions were performed as described previously^30^ using TRIzol. Tissue sections and frozen tissue powder were dissolved in an appropriate volume of TRIzol reagent, while cell culture samples were added to TRIzol as above. cDNA was prepared from 0.8-1ug of RNA, using the RTnanoscript kit (PrimerDesign) with oligo dT and random priming.

**Quantitative PCR**

Primers sets for dystrophin were designed using Primer3 (primer3.ut.ee). PCR products were typically 80-200bp in length, and designed to span one or more introns (where possible) to prevent amplification of genomic DNA. Primers were designed to span exons 1-3 (total dystrophin) and exons 22-23 (unskipped dystrophin). Primers for skipped dystrophin were designed to bind the novel exon 22:24 junction, and exon 25.

Dys Exon1F GTGGGAACAAGTAGAGGACTGTT

Dys Exon3R AGGTCTAGGAGGCGTTTTCC

Dys Exon22F GGAGGAGAGACTCGGGAAAT

Dys Exon23R GTGCCCCTCAATCTCTTCAA

Dys Exon22:24F CTCGGGAAATTACAGAATCACATA

Dys Exon25R TCTGCCCACCTTCATTAACA

Primers to Csnk2a2, Fbxw32, Ap3d1 and GAPDH form part of the geNormPlus kit, and are proprietary property of PrimerDesign.

qPCR runs were performed using Precision SYBR green mastermix (Primerdesign) in white hard-shell 384-well plates (BioRad) using 5-10ng of cDNA per well in a BioRad CFX384 (30ng for dystrophin in cell culture). Cq values were converted to relative quantities (or transcript numbers as described below) and normalized to the geometric mean of 2-3 suitable reference genes (determined by geNorm and Normfinder as described previously^30^); Cell culture: Csnk2a2, Fbxw32 and Ap3d1, Muscle tissue: Csnk2a2, Ap3d1 and GAPDH. Skipping percentages were derived by estimation of absolute transcript numbers, via qPCR analysis using constant amounts of sample cDNAs (or water) mixed with a standard dilution series (10^7^-10^0^ molecules.well^-1^) prepared from purified PCR product of known concentration. As cDNA is predominantly single-stranded an additional cycle is required for parity with PCR products, thus the concentration (in molecules.well^-1^ of standard) at which the dilution series (+cDNA) plateaus corresponds to approximately half the cDNA concentration

## Results

**Intramuscular injection of naked PMO**

IM injection of PMO into the tibialis anterior muscle is a well-established technique for establishing efficacy of targeted exon skipping oligos, and with the M23D PMO this approach routinely generates significant levels of skipped dystrophin mRNA and concomitant dystrophin protein, primarily localized around the injection site. We reasoned that IM injection of skipping oligo alone or in combination with miR31-modulating oligos (at either a 1:1 ratio for ‘protector’ and ‘miR31-analogue’, or 4-fold excess for ‘sponge’) would constitute an appropriate first-pass strategy for establishing the feasibility of a morpholino-mediated microRNA-targeting therapy for DMD.

Initial data encouragingly suggested that addition of ‘sponge’ oligo (skip+sponge) elicited enhanced dystrophin protein (compare fig 2A and B, fig 2C), moreover doing so despite also apparently lowering skipping frequency: exhibiting a pronounced protein/transcript ratio as a consequence (fig 2D). However individual animal-to-animal variation was substantial, thus this increase did not reach significance (indeed, the promising performance of the sponge oligo derived from only two of the six animals in the skip+sponge group). As mean levels of skipped transcript were lower in all combination groups (Fig 2D, top panel), and dramatically so in sponge-treated animals (where sponge oligo was present in a 4:1 ratio with skipping oligo) we reasoned that presence of additional (non-skipping) oligo might compete with skipping oligo for myofibre entry. Efficacy of exon skipping by IM injection is innately variable -being highly dependent on precise location of the needle- and by adding oligo competition we effectively compounded this variability. We thus decided to pursue further studies using our cell culture-based system, reasoning that these would allow us to optimize our treatment protocols more efficiently and cost-effectively.

**Intramuscular injection of exon skipping and miR31-modulating oligos:**
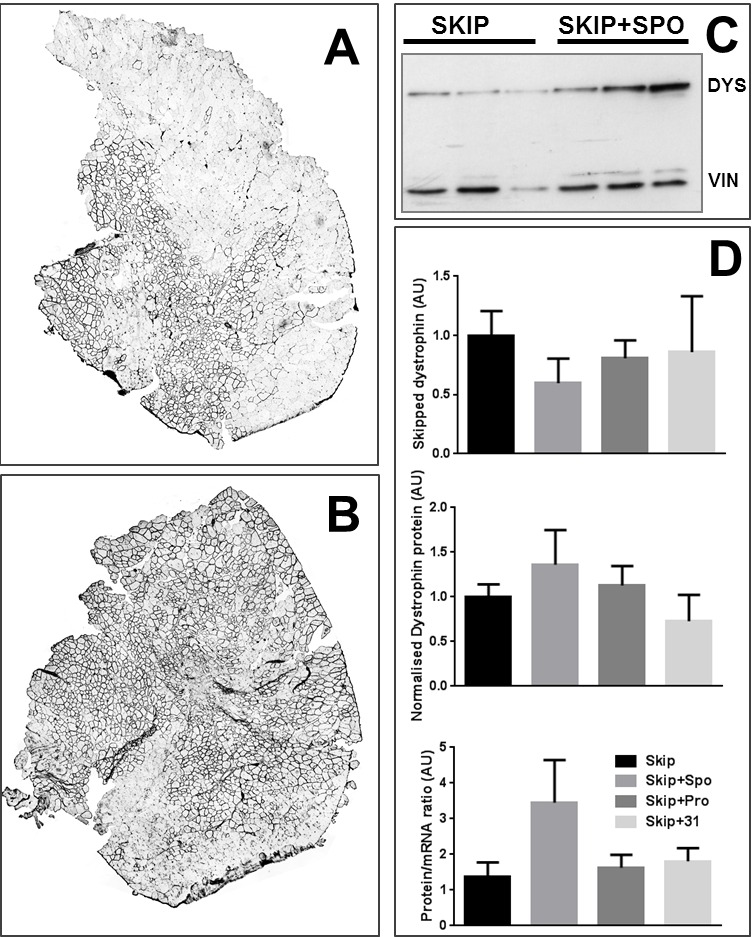
Left panels: Immunostaining for dystrophin restoration in tibialis anterior (TA) muscles injected with M23D skipping PMO alone (A) or with skipping PMO combined with a 4-fold excess of ‘sponge’ PMO (B). Right panel: quantification of dystrophin restoration in injected muscles. Typical western blot for dystrophin restoration in TA muscles treated as indicated (C), qPCR for skipped transcripts, densitometry of western blots for dystrophin protein, and resultant protein.mRNA^-1^ ratio for the treatment groups indicated (D). Means + SEM (N=6). AU: arbitrary units. Dystrophin level normalized to vinculin (protein) or to the geometric mean of three reference genes (mRNA); see methods.

Exon skipping in cell culture

The authors note that establishment of a working cell culture model for measurement of exon skipping at both the transcript and protein level was a non-trivial exercise: for the benefit of researchers wishing to employ a similar system, our methodologies (including unsuccessful nucleofection-based approaches) are below described in full.

**Nucleofection**

Nucleofection of cells with M23D PMO resulted in detectable (but low) levels of skipped transcript (supplementary fig S2), though these levels were insufficient to generate detectable dystrophin at the protein level. Furthermore, co-nucleofection with miR31-modulating oligos reduced skipping levels, presumably again reflecting competition for oligo entry into cells. As nucleofection can only effectively be performed once, and cannot be employed in differentiated myotubes, this approach was abandoned.

**Naked PMO**

Treatment with naked PMO (by simple addition of unconjugated morpholino to the culture medium) in contrast produced skipped transcripts in a clear concentration-dependent fashion, achieving, at higher concentrations, sufficient skipped dystrophin mRNA to generate detectable protein (supplementary fig S2). We have previously shown that dystrophin expression in our cell culture model commences between 50 and 70 hours of differentiation in both dystrophic and wild type cells^30^, thus addition of skipping oligo at ca.50 hours post-differentiation ensures prompt generation of skipped transcripts. Dystrophin is readily detectable at the protein level in wild-type cultures after 90-100 hours (this delay presumably partly reflecting the lengthy 16 hour transcription of dystrophin^31^). In our mdx cell culture model dystrophin protein was not detected until at least 120 hours of differentiation, despite ready detection of skipped transcript at earlier timepoints. This suggests that detectable protein represents the steady accumulation of low levels of highly-stable protein rather than delayed translation. As myotube cultures exceeding 120-144 hours of differentiation demonstrate increasing levels of spontaneous contractile behaviour (resulting in myotube detachment from the culture substrate and thus constituting a necessary experimental endpoint) our data suggests a roughly 48-72 hour window (from dystrophin transcription/skipping to myotube detachment) over which miR31 modulation could be effective.

**miR31-modulation in cell culture**

This above assessment of the timing of dystrophin expression, taken together with the observation that oligonucleotides appear to compete for entry, was used to devise an experimental approach allowing investigation the effects of varying the ratio of miR31-modulating oligos to skipping oligos (either 1:1 or 4:1) and of varying time of delivery of miR31 oligos (either simultaneously with skipping oligo or 24/48 hours afterward: see schematic, supplementary fig S3): miR31 modulation would be expected to demonstrate obligate dependence on exon skipping, thus can potentially be delayed until sufficient skipped transcripts have been generated.

As shown in fig 3, only simultaneous addition of protector oligo at 1:1 produced significant increases in protein:mRNA ratio over skipping oligo alone (compare fig 3A with 3B and 3C), with all other treatments being equivalent to skip alone, or eliciting a reduction in ratio. Note however regardless of time of addition, presence of miR31-modulating oligos resulted in lower overall levels of skipped transcript in both protector and 31-analogue (with higher levels in sponge-treated samples likely stemming from increased dystrophin transcription –see below). Our data nevertheless suggests a biological role for all three modulating oligos, as treatment with sponge oligo consistently raised overall dystrophin transcription levels, without demonstrable effect on protein/mRNA ratio (supplementary fig S4), while miR31 oligo unexpectedly appears to lower dystrophin translation, leading to significant decrease in protein:mRNA ratio (fig 3C).

**miR31-modulation in a cell culture model of exon skipping:**
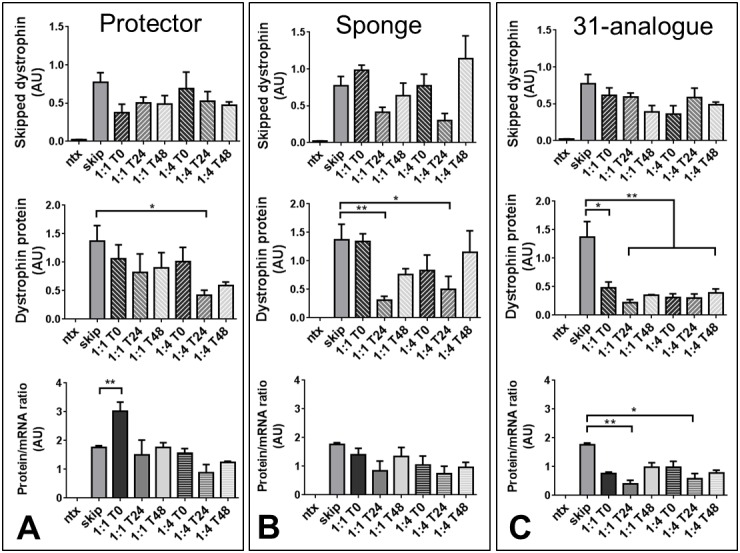
Skipped dystrophin transcripts (top), dystrophin protein (middle) and resultant protein.mRNA^-1^ ratio (bottom) in dystrophic myotube cultures carrying the mdx mutation (H2KSF1), either untreated (ntx), incubated with M23D skipping oligo alone (skip), or with M23D combined with miR31-modulating oligos (as indicated) added either simultaneously (T0), 24 hours after M23D addition (T24) or 48 hours after (T48). miR31-modulating oligos were added at 1:1 or 4:1 ratios with skipping oligo as indicated. Means + SEM (N=3) *=P<0.05, **=P<0.01. AU: arbitrary units. Dystrophin level normalized to vinculin (protein) or to the geometric mean of three reference genes (mRNA); see methods.

**miR31-modulation via Vivo-Morpholino**

The cell culture data above suggested that a simultaneous 1:1 skip+protector combination constituted a viable approach: as the most dystrophin-specific modulating oligo, protector-based strategies are also the most therapeutically attractive. It was therefore decided to take this approach forward to in vivo study in the *mdx* mouse. The high variability of our pilot in vivo investigation (intramuscular injection of naked PMO) demonstrated that this local delivery method is inappropriate, therefore for these further studies we decided to employ systemic delivery using Vivo-Morpholinos. Unexpectedly, intravenous delivery of M23D skipping Vivo-Morpholino proved to be universally fatal, with all treated mice apparently beginning to recover normally from isoflurane anaesthesia before abruptly becoming unresponsive (see discussion).

Our Vivo-Morpholino studies therefore utilized an intraperitoneal delivery route. Our preliminary trial using IP delivery of vivo-skipping oligo demonstrate relatively high levels of skipped transcript and protein in muscle tissues exposed to the peritoneal cavity, namely the diaphragm and the body wall musculature, therefore these tissues were selected for analysis. Skipped transcripts were detectable in other muscle tissues studied (quadriceps, TA, triceps, EDL, soleus) but were 1-2 orders of magnitude lower than levels in diaphragms and body walls, and did not result in demonstrable dystrophin protein (supplementary fig S5).

Mice were thus treated via IP injection with skipping oligo alone, or in 1:1 combination with protector oligo or a ‘scrambled’ protector sequence. As shown in fig 4A, greater quantities of skipped transcript were achieved (and achieved more consistently across treatment groups) in the diaphragm than in the body wall, though in contrast to our naked PMO data, the presence of additional non-skipping oligo -protector or scramble- unexpectedly appeared to potentiate uptake (albeit variably) in body wall. Expression of dystrophin is 30-50% higher in dystrophic diaphragm than body wall (supplementary fig S6) thus the higher levels of skipping achieved in diaphragms could conceivably be simply due to higher levels of transcripts available for skipping. However, even when expressed as a percentage of total dystrophin, diaphragm skipping percentages are consistently around 20% across treatment groups, while body walls treated with skipping oligo alone remain around 10% (as in our pilot study, supplementary fig S5), increasing to roughly 20% in the presence of additional non-skipping oligo (table 1). This phenomenon was also observed with combined Vivo-Morpholinos in cell culture (supplementary fig S7).

**Intraperitoneal delivery of exon skipping and miR31-modulating Vivo-Morpholinos to diaphragms and body wall musculature:**
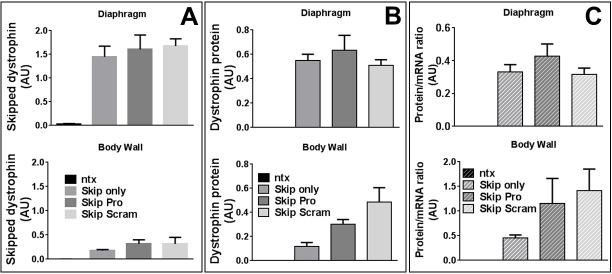
Skipped dystrophin transcripts (A), dystrophin protein (B) and resultant protein.mRNA^-1^ ratio (C) in diaphragm (top) and body wall (bottom) musculature, treated with Vivo-Morpholinos (as indicated). NTX: no treatment; Skip only: M23D skipping oligo alone; Skip Pro: M23D combined with protector; Skip Scram: M23D combined with scrambled protector sequence. Means + SEM (N=6). AU: arbitrary units. Dystrophin level normalized to vinculin (protein) or to the geometric mean of three reference genes (mRNA); see methods.

**Table 1: Skipping percentages following Vivo-Morpholino treatment:** Skipped dystrophin transcripts as a percentage of total dystrophin transcripts in diaphragms and body walls from mdx mice treated with Vivo-Morpholinos (as indicated). NTX: no treatment; Skip only: M23D skipping oligo alone; skip+pro: M23D combined with protector; skip+scram: M23D combined with scrambled protector sequence. Means + SEM (N=6 –same sample groups as in figure 4)

	Body wall (%)	Diaphragm (%)
NTX	0.43±0.1	1.1±0.1
Skip Only	8.9±1.3	21.6±2.5
Skip + Pro	18.0±3.9	21.8±1.8
Skip + Scram	20.5±5.3	24.0±1.9

Note that, as expected, the generation of stable (skipped) mature dystrophin transcripts leads to a mild increase in total dystrophin transcripts in both tissues in treated animals (supplementary fig S6).

When examined at the histological level for presence of sarcolemmal dystrophin (figs 5 and 6), all three treatment groups were readily distinguished from untreated muscle, but could not be further separated into individual treatments: skipping oligo alone or combined with protector/scrambled oligo produced comparable quantities of widely-distributed dystrophin-positive fibres. While overall levels varied somewhat from animal to animal this was not associated with any particular treatment. Western blotting analysis revealed dystrophin levels (normalized to the muscle membrane marker vinculin) similarly varied substantially from sample to sample (fig 4B), and no significant differences were observed between treatment groups in diaphragm dystrophin levels. In body walls both scramble- and protector-treated tissue (mirroring the higher levels of successfully skipped mRNA described above) showed higher mean protein than “skip only” samples: an intriguing result but clearly not one restricted to (or dependent upon) “protector” sequence.

**Dystrophin expression in body walls following intraperitoneal delivery of exon skipping and miR31-modulating Vivo-Morpholinos:**
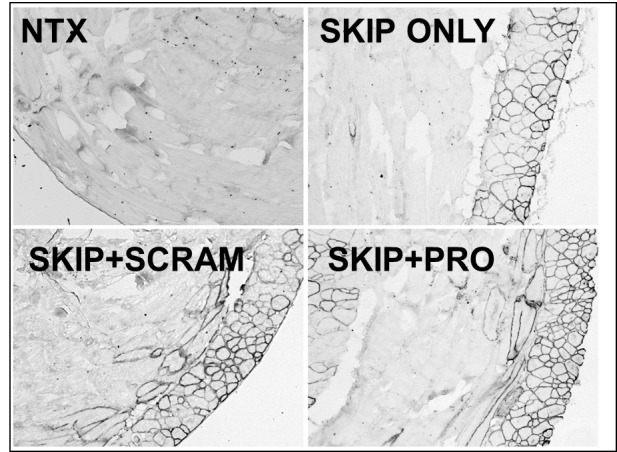
Immunostaining for dystrophin restoration in body wall cryosections treated as indicated. NTX: no treatment; SKIP ONLY: M23D skipping oligo alone; SKIP+PRO: M23D combined with protector; SKIP+SCRAM: M23D combined with scrambled protector sequence. Note that body wall architecture has two closely-associated muscle groups lying perpendicular to each other: Peritoneum-proximal tissue is transversely sectioned here, while distal tissue is longitudinal.

**Dystrophin expression in diaphragms following intraperitoneal delivery of exon skipping and miR31-modulating Vivo-Morpholinos:**
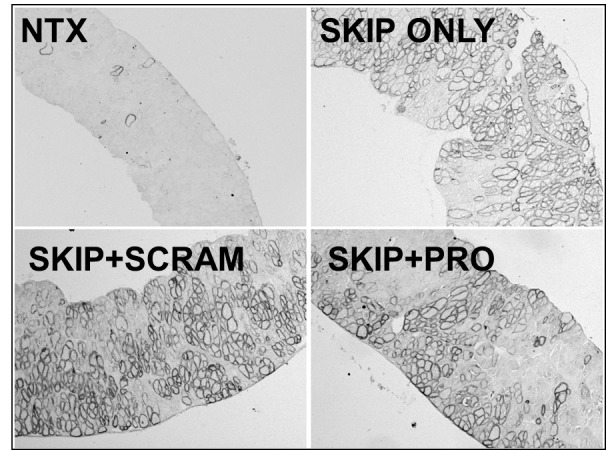
Immunostaining for dystrophin restoration in diaphragm cryosections treated as indicated. NTX: no treatment; SKIP ONLY: M23D skipping oligo alone; SKIP+PRO: M23D combined with protector; SKIP+SCRAM: M23D combined with scrambled protector sequence.

Ultimately (as shown in fig 4C) when assessed as a ratio of protein (as determined by western blot) to mRNA (as determined by qPCR), no significant differences were detected between treatments in either tissue studied.

## Discussion

A therapeutic agent capable of driving increased dystrophin translation would be a highly attractive discovery: acting at the post-transcriptional level, such an agent could theoretically act synergistically with all exon skipping approaches, regardless of the specific mutations in question (and indeed also be of benefit to patients producing viable but insufficient levels of dystrophin mRNA). If such an agent could be employed using existing oligonucleotide chemistry for which a substantial body of toxicology and pharmacokinetic data already exists, this would greatly expedite transition from a laboratory context to a therapeutic one. For these reasons we decided to build on the work of Cacchiarelli *et al* 21 and investigate the potential of modulating the microRNA miR31 using morpholino oligomers in a dystrophic context. As we here show (despite encouraging data from our cell culture model) under the conditions selected, modulation of miR31 via PMO appears to be of no significant benefit to our *in vivo* model, the *mdx* mouse. Were it not for our cell culture data, it would be tempting to take the parsimonious interpretation: that morpholino-based modulation of miR31 simply does not work. It is important to recognize, however, that our *in vivo* analysis is not exhaustive: due to practical restrictions, large scale studies similar to those used in cell culture (multiple doses, staggered administrations) were not carried out in our animal model, and the unexpected lethality of IV-administered Vivo-Morpholino precluded investigation of miR31 modulation in a more systemic context. This work also necessarily operated under several significant restrictions, which we discuss below.

The primary difficulty with this work is oligonucleotide entry: as suggested by both intramuscular injections and cell culture treatments with nucleofected PMO, oligo uptake appears to be both comparatively low and moreover, readily saturable: inclusion of additional oligonucleotides alongside the skipping oligo M23D is here repeatedly shown to lower overall levels of exon skipping. As levels of skipped transcripts otherwise correlate well with M23D concentration in cell culture (supplementary figure S2) the logical interpretation is that multiple oligonucleotides compete for cell entry, most likely via an endocytotic process^32^^,^ 33. As a consequence of this competition, levels of skipped transcript and subsequent dystrophin protein as measured here were frequently lower following co-treatment with miR31-modulating oligos, hence our assessment of miR31 modulation by a ratiometric (protein per transcript) rather than an absolute (total protein) approach. While the authors acknowledge that this competitive effect could itself be considered to present a significant barrier to therapies based on multiple oligos, such direct competition could presumably be readily circumvented by staggered oligo administration. Moreover, as much of the focus in oligonucleotide therapies is now moving toward enhancing cellular uptake of these molecules, saturation of oligo uptake (carrier-mediated or not) is unlikely to constitute a long-term impediment.

An additional complication with assessing the potential of oligonucleotide therapies is that as small uncharged (and unlabelled) molecules, oligo uptake is non-trivial to quantify. Uptake of skipping oligo can be assessed -at least in relative terms- by measuring levels of exon skipping; however within this study the extent of uptake of miR31-modulating oligos is essentially unknown. As demonstrated in our pilot IM injection studies, uptake is also inherently somewhat variable: the combination of two variable and mutually-competitive oligonucleotide uptakes, only one being (relatively) quantifiable, presents a significant barrier to data interpretation, and highlights the importance of sufficient sample numbers.

Application of naked PMO directly to differentiating myotubes in a cell culture system allows us to partially circumvent the issue of oligonucleotide competition: unlike *in vivo* scenarios where oligo not sequestered in cells is rapidly cleared via the circulatory system, here oligos remain trapped in the culture medium, increasing the effective dose delivered. If uptake is indeed carrier-mediated, this should allow intracellular oligonucleotide to reach equilibrium with the external media, regardless of total oligo concentration or number of different oligo species used. Under such a system, staggered administration of oligos would more accurately expected to represent a “head-start” for exon skipping. Unexpectedly, oligo combinations (both co-administered and delivered in a staggered fashion) consistently resulted in lower levels of skipped transcripts: either indicating that equilibrium had not been achieved even over the lengthy course of the experiment (implying a very slow rate of carrier-mediated uptake), or suggesting the presence of additional intracellular processes for which oligos compete (for example, subcellular trafficking –a non-trivial concern in the highly-structured intracellular environment of multinucleated myotubes). These observations accepted, our cell culture data suggests that all three miR31-modulating oligos are able to exert some biological effect (albeit not always a beneficial one). While having no apparent effect on protein:mRNA ratio, ‘Sponge’ oligo nevertheless appears to elicit a minor (but consistent) increase in dystrophin transcription (supplementary fig S4) –the most plausible explanation being that the ‘sponge’ sequence, as a direct miR31-chelating agent, influences all miR31-associated processes including those moderating commitment to, and progression through, myogenesis (such as translation of Myf5^25^). In effect, myotubes from cultures treated with ‘sponge’ may proceed along the myogenic program at a moderately accelerated rate. While this could be considered to be of some small therapeutic use, miR31 is also implicated in the regulation of a large number of cellular processes throughout the body, including tumour suppression^34^: as a global miR31-targeting agent, a ‘sponge’ approach would be expected to carry significant side-effect burden, obviating use of this strategy outside of a commensurate level of therapeutic benefit.

Intriguingly, our miR31 analogue appears to actively reduce dystrophin expression at the protein level (fig 3C): we presently are unable to explain this phenomenon: a morpholino corresponding to the miR31 sequence should be incapable of forming a viable repressor complex with Dicer and Argonaute proteins, yet still be capable of interfering with miR processing (as outlined in Fig1). The reverse complement of miR31 (miR31*) may possess biological activity (though data supporting this is minimal^35^), under which scenario our morpholino might be expected to act as a miR31* ‘sponge’ (with the added complication of potentially sequestering degradation machinery due to full sequence complementarity). However, a reduction in dystrophin transcription as a consequence of miR31* blockade would imply diametrically opposed roles for miR31 and its complement, which seems biologically implausible.

Recently published work (as with Cacchiarelli* et al* 21) from the group of Irene Bozzoni^36^ however suggests a more complex role for the miR31 locus, with this region generating both miR31 and a long non-coding RNA (lnc-mir31) via mutually exclusive pathways. While both miR31 and lnc-miR31 are proposed to play roles in control of myogenic commitment and progression, our miR31 analogue PMO might perturb the ratio of these two ncRNAs, with significantly reduced dystrophin translation as a consequence.

Finally, our cell-culture system revealed that ‘protector’ PMO exerted no effect on overall dystrophin transcription (as expected), instead slightly reducing levels of skipped transcript (as common to all three miR31-modulating oligos) but also reproducibly increasing the yield of protein per transcript when delivered simultaneously at a 1:1 ratio with skipping oligo. Failure to observe similar increases when using higher ratios (4:1) or when delivered 24 or 48 hours after skipping oligo are troubling, but may reflect the delicate balance of opposed factors involved: earlier addition (or higher concentration) of ‘protector’ oligo reduces total skipped transcripts, while later addition of ‘protector’ limits total exposure of skipped transcripts to this oligo before the necessary experimental endpoint (onset of spontaneous myotube contraction). Our data thus suggested that ‘protector’ based strategies might be effective in enhancing dystrophin translation, presenting a viable avenue to take forward to further investigation.

As described above, for our final *in vivo* study we elected to compare efficacy of skipping oligo alone versus skipping oligo combined with either protector oligo or a scrambled protector sequence (see supplementary fig S1). We reasoned that inclusion of a scrambled sequence should more readily allow identification of protector-specific effects even if overall skipping levels were lowered. We further reasoned that use of Vivo-Morpholinos might circumvent many of the oligo uptake concerns described above. These commercially-available morpholinos carry an octa-guanidine dendrimer, and this moiety confers independently cell-penetrant properties on the conjugated morpholino, circumventing saturation of slow carrier-mediated uptake processes and moreover permitting systemic delivery. As described here, however, systemic intravenous delivery of a Vivo-Morpholino of the M23D skipping sequence was invariably fatal within minutes, with a crude post-mortem suggesting systemic blood clotting as the cause. Why this should be the case is not clear: although sufficiently hepatotoxic to be inappropriate in a human therapeutic context, intravenous use of these cell-penetrant dendrimer-conjugated oligos as exon-skipping agents has previously been shown to drive systemic restoration of dystrophin in the *mdx* mouse^37^^,^ 38. Similar fatalities reported by Ferguson* et al* the same month our studies were performed^39^ led those authors to propose that oligo dimerisation may play a role, effectively doubling the local concentration of the dendrimer moiety, resulting in adverse interactions with clotting factors. The potential for self-dimer formation in M23D is limited however, and attempts to disrupt any possible multimers prior to injection (sonication, vortexing, heating and crash-cooling) had no effect on the toxicity of this treatment. Moreover, as noted the M23D sequence has previously been shown to be highly effective when employed as a Vivo-Morpholino, thus our experiences are puzzling. Given these findings, this approach was abandoned (it was judged unlikely that combination of M23D with miR31-modulating Vivo-Morpholinos would alter the lethality of intravenous delivery), and the authors would advise caution to any other investigators considering systemic use of Vivo-Morpholinos. Our alternative strategy (intraperitoneal administration) was well-tolerated however, resulting in widespread dystrophin restoration in the diaphragm muscle (fig 6), and adequate restoration in peritoneum-proximal body wall musculature (though as noted, our data suggests systemic penetrance of oligo was poor: dystrophin protein was effectively absent in limb muscle tissues, and limited even in distal body wall: fig 5).

Skipped transcripts and skipping percentages (skipped transcripts as a fraction of total dystrophin transcripts) were relatively consistent in diaphragms regardless of treatment (ca. 20%, see table 1), while levels in body walls were lower and more variable. Interestingly, co-administration of protector or scrambled sequence both resulted in higher mean levels of skipped transcripts in body walls, effectively the converse of our observations with naked PMO. Given mouse posture with respect to the peritoneal cavity, IP-administered morpholino might reasonably be expected to pool proximal to the body wall, thus duration of exposure to Vivo-Morpholino may be greater in this tissue. It is possible that the charged octa-guanidine conjugates act on membranes in a concentration-dependent fashion: under prolonged exposure to Vivo-Morpholino, presence of additional dendrimer moieties may facilitate uptake of PMO regardless of conjugated oligomer sequence. Supporting this hypothesis, incubation of cultured myotubes with Vivo-Morpholinos (analogous to proximal pooling of oligo) exhibited the same phenomenon: M23D combined with either protector or scrambled sequence at 500nM oligo consistently led to higher skipping levels than M23D alone (supplementary fig S7). The authors note that overall levels of skipping were lower and more variable in body walls, however, thus this *in vivo* observation may simply be an artefact. Unlike diaphragms, where the entire muscle bulk could be readily identified and collected, the body wall strips used for this analysis represent only a portion of the overall body wall musculature. While all effort was taken to collect a consistent quantity of tissue from the same region of the body wall, slight variations are essentially unavoidable.

As described above, the chief findings of our Vivo-Morpholino work were that (despite variable levels of skipping and protein expression) no significant differences in protein:mRNA ratios were observed in either muscle tissue in any of our treatment groups. These findings are disappointing: uptake of M23D skipping oligo appears to be reasonably high and unperturbed (indeed possibly even potentiated) by co-administration of protector or scrambled oligos, thus it seems reasonable to assume that both protector and scrambled oligos were taken up to a similar extent. In terms of minimizing the caveats listed herein this approach appears highly successful, yet reveals our protector-based strategy to be of no apparent benefit whatsoever under these conditions. It should be noted that while exon skipping works at the level of transcript splicing, a protector approach necessarily applies at the translational level. One molecule of skipping oligo can act repeatedly, splicing multiple transcripts; conversely, protector binds 1:1 with skipped transcripts thus one molecule of oligo would presumably protect only one transcript from miR31-mediated translational blockade at a time. Taken together with our cell culture data (where substantially lower levels of transcripts are involved), the failure of this approach at the *in vivo* level suggests that a level of protector oligo sufficient for efficacy in the *mdx* mouse was not achieved. As noted, due to time and resource restrictions (and the unexpected lethality of systemic administration) we were unable to perform more comprehensive investigations in our mouse model, such as a staggered administration approach. An attractive line for future studies would be use of the *mdx* ^3Cv^ mouse: this mouse exhibits a low (and consistent) level of endogenous skipping^40^^,^ 41, thus would allow study of miR31-blocking oligos in isolation, free from the additional variable of skipping efficiency.

In conclusion, it could reasonably be argued that if higher oligo doses are required for miR31-modulation, such doses might well be better reserved for exon skipping in the first place. While miR31-modulation potentially offers a universally-applicable therapeutic for DMD, the levels of oligo ostensibly required (as shown here) would unfortunately seem to render this approach impractical under current oligonucleotide chemistries. A recent study (published after the work described here) further showed that several microRNAs may jointly contribute to suppression of dystrophin translation in BMD, including miR146b and miR374a^22^. MiR31-modulation alone may thus be insufficient in vivo, necessitating PMO-mediated modulation of two or more additional miRs to relieve the translational block. Given that achieving effective delivery of even a single species of oligonucleotide is challenging, such a requirement would further argue strongly against the feasibility of this approach.

## Appendix

**Figure:**
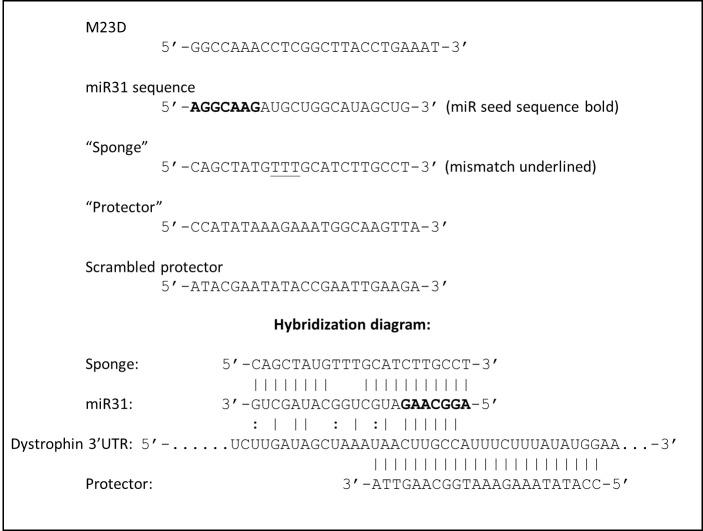
**Supplementary figure S1:** Oligonucleotide sequences and hybridization scheme. Sequences for skipping oligo (M23D), miR31, ‘sponge’ and ‘protector’ oligos (miR31 analogue oligo is simply miR31 sequence, substituting T for U, as morpholinos do not use uracil)

**Figure:**
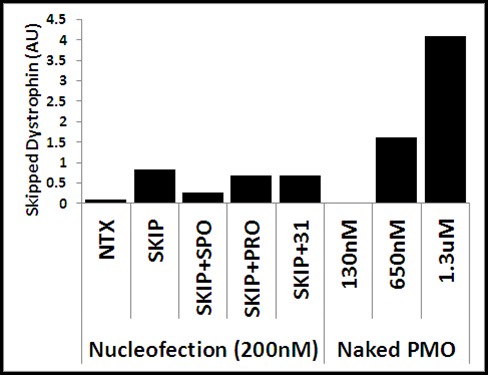
**Supplementary figure S2: Exon skipping in cell culture** Levels of skipping transcript detected in cells differentiated after nucleofection with M23D and miR31-modulating oligos (leftmost 5 columns), or treated with naked PMO added directly to culture wells at the concentrations indicated (rightmost 3 columns). NTX: no treatment; SKIP: M23D skipping oligo alone; SKIP+SPO: M23D combined with fourfold excess of sponge oligo; SKIP+PRO: M23D combined with equimolar protector; SKIP+31: M23D combined with equimolar miR31-analogue sequence. AU: arbitrary units. Dystrophin mRNA level normalized to the geometric mean of three reference genes; see methods.

**Figure:**
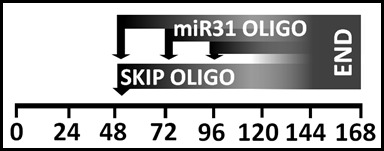
**Supplementary figure S3: schematic for exon skipping in cell culture** Proliferating H^2K^SF1 cells are transferred to differentiation medium (T0). M23D skipping oligo is added between 48 and 52 hours following a partial media change (50% replacement) and no further media changes occur. MiR31-modulating oligos are added either simultaneously, or after 24 or 48 hours. Beyond 120-144 hours of differentiation, onset of spontaneous contractile behaviour necessitated termination of culture and sample collection.

**Figure:**
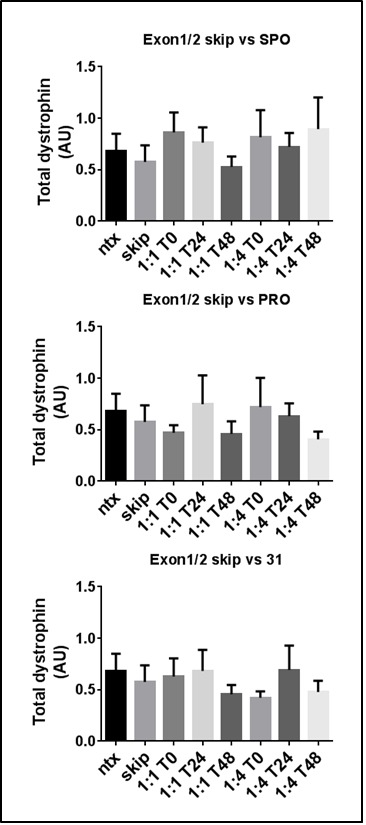
**Supplementary figure S4: Total dystrophin expression in cell culture following miR31-modulation**. Total dystrophin transcripts in dystrophic myotube cultures carrying the *mdx* mutation (H^2K^SF1), either untreated (ntx), incubated with M23D skipping oligo alone (skip), or with M23D combined with miR31-modulating oligos (as indicated. Top: sponge. Middle: protector. Bottom: miR31-analogue) added either simultaneously (T0), 24 hours after M23D addition (T24) or 48 hours after (T48). miR31-modulating oligos were added at 1:1 or 4:1 ratios with skipping oligo as indicated. Means + SEM (N=3). AU: arbitrary units. Dystrophin mRNA level normalized to the geometric mean of three reference genes; see methods.

**Figure:**
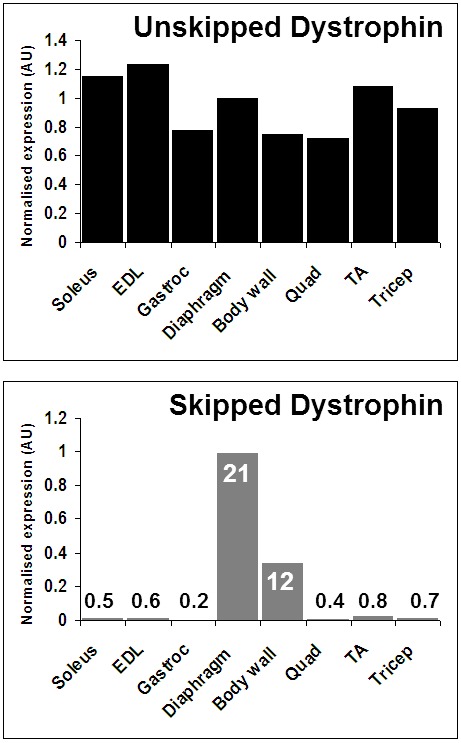
**Supplementary figure S5: Exon skipping in mice following IP administration of M23D Vivo-Morpholino**. Unskipped (top) and skipped (bottom) dystrophin transcripts in 8 muscle groups (soleus, extensor digitorum longus, gastrocnemius, diaphragm, body wall, quadriceps, tibialis anterior and triceps, as indicated), 2 weeks after a single IP injection of M23D Vivo-Morpholino. Numbers in lower chart: percentage skipped transcripts (skipped transcripts as a percentage of total dystrophin transcripts). AU: arbitrary units. Dystrophin mRNA level normalized to the geometric mean of three reference genes; see methods.

**Figure:**
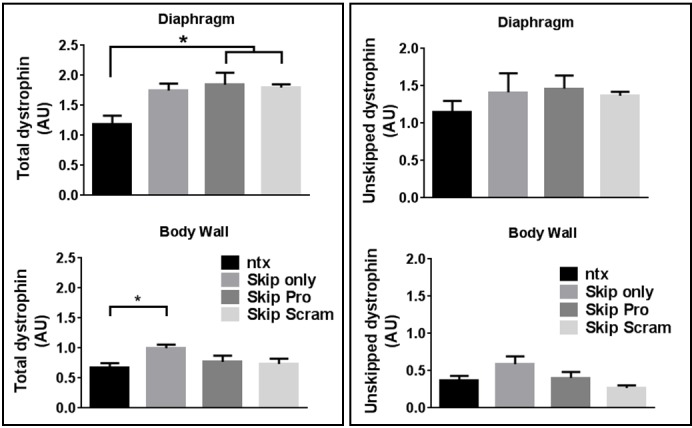
**Supplementary figure S6: Intraperitoneal delivery of exon skipping and miR31-modulating Vivo-Morpholinos to diaphragms and body wall musculature**. Total dystrophin transcripts (left panel), and unskipped dystrophin transcripts (right panel) in diaphragm (top) and body wall (bottom) musculature, treated with Vivo-Morpholinos (as indicated). NTX: no treatment; Skip only: M23D skipping oligo alone; Skip Pro: M23D combined with protector; Skip Scram: M23D combined with scrambled protector sequence. Means + SEM (N=6). AU: arbitrary units. Dystrophin mRNA level normalized to the geometric mean of three reference genes; see methods.

**Figure:**
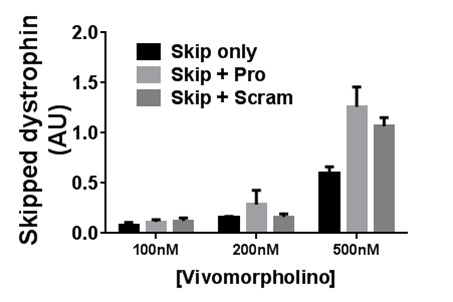
**Supplementary figure S7: Exon skipping via M23D and miR31-modulating Vivo-Morpholinos in cell culture**. Skipped dystrophin transcript levels from dystrophic (H2KSF1) myotube cultures incubated with Vivo-Morpholinos at the concentrations indicated for 72 hours. Minimal skipping was detected at concentrations below 500nM; however at 500nM, presence of additional (non-skipping) oligo unexpectedly potentiates exon skipping. Skip only: M23D skipping oligo alone; Skip+Pro: M23D combined with protector; Skip_Scram: M23D combined with scrambled protector sequence. Means + SEM (N=3). AU: arbitrary units. Dystrophin mRNA level normalized to the geometric mean of three reference genes; see methods.

## Data

All data used to generate the figures shown is freely available under Creative Commons licence at the figshare link below.

https://figshare.com/articles/MiR31_in_DMD_raw_data_xls/3102766

## Competing Interests

JCWH declares no competing interests exist.

DJW is on the Scientific Advisory Board of Akashi Therapeutics, a company developing treatments for Duchenne muscular dystrophy. DJW is also a member of the Treat-NMD Advisory Committee for Therapeutics that provides confidential guidance on the translation and development path of therapeutics programs in rare neuromuscular diseases. This does not alter the authors' adherence to all PLOS policies on sharing data and materials.
